# Phylogenetic characterization of *Salmonella enterica* from pig production and humans in Thailand and Laos border provinces

**DOI:** 10.14202/vetworld.2019.79-84

**Published:** 2019-01-16

**Authors:** Rangsiya Prathan, Asinamai Athliamai Bitrus, Nuananong Sinwat, Sunpetch Angkititrakul, Rungtip Chuanchuen

**Affiliations:** 1 Research Unit in Microbial Food Safety and Antimicrobial Resistance, Department of Veterinary Public Health, Faculty of Veterinary Science, Chulalongkorn University, Bangkok 10330, Thailand; 2 Department of Farm Resources and Production Medicine, Faculty of Veterinary Medicine, Kasetsart University, Kamphaengsean campus, Nakhonpathom, 73140 Thailand; 3 Research Group for Prevention Technology in Livestock, Faculty of Veterinary Medicine, Khon Kaen University, Khon Kaen 40000, Thailand

**Keywords:** Laos, pig, *Salmonella*, Thailand

## Abstract

**Background and Aim:**

The genetic relationship among serotypes of *Salmonella* *enterica* from food animals, food of animal origin, and human is of interest as the data could provide an important clue for the source of human infection. This study aimed to determine the genetic relatedness of *S. enterica* from pig production and human in Thailand–Laos border provinces.

**Materials and Methods:**

A total of 195 *S. enterica* serotypes isolated from pig and pork (n=178) and human (n=17) including four serotypes (Typhimurium, Rissen, Derby, and Stanley) were randomly selected to examine their genetic relatedness using highly conserved sequence of three genes (*fim* A, *man* B, and *mdh*).

**Results:**

The results showed that 195 *Salmonella* isolates of four different serotypes were grouped into five different clusters, and members of the same *Salmonella* serotypes were found in the same cluster. *Salmonella* isolated from pig production and human in Thailand–Laos border provinces represented overlapping population and revealed a high degree of similarity, indicating close genetic relationship among the isolates.

**Conclusion:**

The results support that the determination of *Salmonella* serotyping combined with analysis of phylogenetic tree can be used track the clonal evolution and genetic diversity of *Salmonella* serotypes in different host species.

## Introduction

Due to the regional economic integration (known as ASEAN Economic Community), rapid growth of cross-border activities between Thailand and their regional neighbors is expected. These cross-border activities including the movement of people, goods, and services, in either legal or illegal manners, could lead to greater risk of cross-border transmission of infectious diseases, especially foodborne pathogens [1]. Thailand and Laos share a common border of approximately 1800 km in length with up to 36 crossing points [2]. Livestock, such as pigs and cattle, are common commodities [3],movement and unhygienic slaughter of livestock occur in and around Thailand–Lao PDR border. Thus, causing major concern among public health authorities on either side of the border. Pigs are smuggled in and out between Thailand and Laos every day, and these animals are illegally slaughtered without recourse to best hygienic practices [4]. Therefore, these could be a critical source for spreading of foodborne pathogens.

Salmonellosis represents a growing threat to global public health. It was estimated that Salmonellosis accounts for about 155,000 deaths and 9,380,000 infections in humans worldwide [5]. *Salmonella* surveillance program using serotyping for subtyping and strain differentiation has been reported [6]. This technique examined *Salmonella* O and H antigen based on immune reaction technique. Even if around 2500 *Salmonella* serotypes can be differentiated, this technique has limited value in determining the genetic relationship of strains within the same or different serotype [7]. More discriminatory subtyping methods, such as phage typing and pulsed-field gel electrophoresis, are thus commonly used to subtype *Salmonella*, particularly as part of national and international salmonellosis surveillance systems [8,9].

Information on the main food animal reservoirs for *Salmonella* and the modes of transmission are extremely limited in Thailand.

This study was conducted to gain a better understanding of the ability of *Salmonella* to be transmitted to humans from pigs. Therefore, this study aimed to determine the genetic relatedness of *Salmonella enterica* isolates obtained from pig production and human in Thailand–Laos border provinces.

## Materials and Methods

### Ethical approval

Research protocols involving human subjects were approved by the Ethics Committee of the Faculty of Medicine, Khon Kaen University (the authorization ID, HE572136).

### Samples collection

A total of 195 *S. enterica* isolates collected from pig and pork (n=178), and human (n=17) were selected and used in this study. All isolates were obtained from samples collected in three provinces in Thailand including Nong Khai, Mukdahan, and Ubon Ratchathani and two provinces in Laos including Vientiane and Savannakhet (Table-1). The study locations included one municipal pig slaughterhouses, one municipal fresh market, and one municipal hospital in each province. These slaughterhouses and markets were selected so that the animals could be tracked from the slaughterhouses to the markets. The samples were collected 3 times with 3-4-month interval from each sampling site. Due to logistical constraints, random sampling was not possible. The fecal samples were collected from pigs by rectal evacuation after bleeding but before the scalding process. Carcass samples were collected before delivery to the market by swabbing an area of approximately 50 cm^2^ on each carcass using sterile gauze. The same pigs were tracked and sampled again at fresh markets. At fresh markets, the samples were collected from pig carcasses by swabbing with gauze at the corresponding retail shop.

**Table-1:** Number of *Salmonella* isolates examined in this study (n=195).

Sample type	Number of *Salmonella* isolates					Total

	Thailand			Laos		
	
	NK	MH	U	VT	SV	
Pig	11	11	6	13	19	60
Pig carcasses	10	11	7	10	20	58
Pork	12	9	9	20	10	60
Human	3	9	0	2	3	17
Total	36	40	22	45	52	195

NK=Nong Khai, MH=Mukdahan, U=Ubon Ratchathani, VT=Vientiane, SV=Savannakhet

Human stool samples were collected from workers at slaughterhouses, butchers, and workers at a retail meat shop in fresh markets and patients with diarrhea at hospitals. The slaughterhouse workers, butchers, and retail meat workers were requested to provide their own stool samples. Nurses or technicians collected samples from patients. Due to a low number of possible human samples, efforts were made to obtain most possible participants.

### Salmonella isolation and identification

The *Salmonella* strains were isolated using the standard protocol ISO6579:2002 (E). Three colonies were initially collected from each positive sample and subjected to serotyping by slide agglutination based on the Kauffman–White scheme at the Center of Antimicrobial Resistance in Foodborne Pathogens (in cooperation with the World Health Organization), Faculty of Veterinary Science, Chulalongkorn University.

### Polymerase chain reaction (PCR) and DNA sequencing

All *Salmonella* isolates were subjected to molecular typing based on *fimA, manB*, and *mdh* genes with specific primer pairs as reported [10]. PCR reactions were carried out in 25 μL reaction volume containing 12.5 μL of 2× Reddymix™ PCR Master Mix, 1 μL of each forward and reverse primers, 5 μL of DNA template, and 5.5 μL of sterile distilled water. Amplification conditions for *manB*, *fimA*, and *mdh* gene targets were as follows: Initial denaturation at 95°C for 9.5 min, followed by 40 cycles of 95°C for 45 s, annealing for 45 s at either 55°C *(man*B) or 58°C *(fimA* and *md*h), extension at 72°C for 1 min, and a final extension step of 7 min at 72°C. Amplified PCR products were analyzed by electrophoresis through 1.2% agarose gels. All PCR products were purified using Nucleospin^®^ Gel and PCR clean up (Macherey-Nagel, Düren, Germany) and submitted for sequencing (First Base Laboratories, Selangor Darul Ehsan, Malaysia). All sequences were assembled and proofread using SeqMan and aligned using the MUSCLE [11] in MEGA 6.0.

### Phylogenetic analyses

DNA sequence alignments of *fimA, manB*, and *mdh* were used for the construction of phylogenetic trees. The phylogenetic tree was built using the neighbor-joining (NJ) method. The evolutionary history was inferred using the NJ method [12]. The evolutionary distances were computed using the Maximum Composite Likelihood method [13] and are in the units of the number of base substitutions per site. The analysis involved 195 nucleotide sequences. All positions containing gaps and missing data were eliminated. There were a total of 1989 positions in the final dataset. Evolutionary analyses were conducted in MEGA6 [14].

## Results and Discussion

Salmonellosis presents a significant public health challenge due to the number of cases per year and due to the ease of transmission to humans through the food chain [15]. Molecular typing methods that utilize the sequences of highly conserved housekeeping and virulence gene offer a robust and versatile approach for the detection of foodborne salmonellosis. The technique helps to accurately identify routes of transmission and sources of infection, especially in the detection of bacterial population (*Salmonella* Enteritidis and *Salmonella* Typhimurium) with a very large clonal lineage and limited heterogeneity [16]. In addition, despite the extensive strategy invested in surveillance, the burden of foodborne Salmonellosis due to *S. enterica* in humans is on the increase. Moreover, since *S. enterica* serotypes are made up of several sublineages with divergent antigenic representation, antimicrobial resistance phenotypes, and virulence. Understanding the evolution of these species is crucial in identifying sources, routes of transmission as well asprediction and prevention of outbreaks [17].

**Figure-1:**
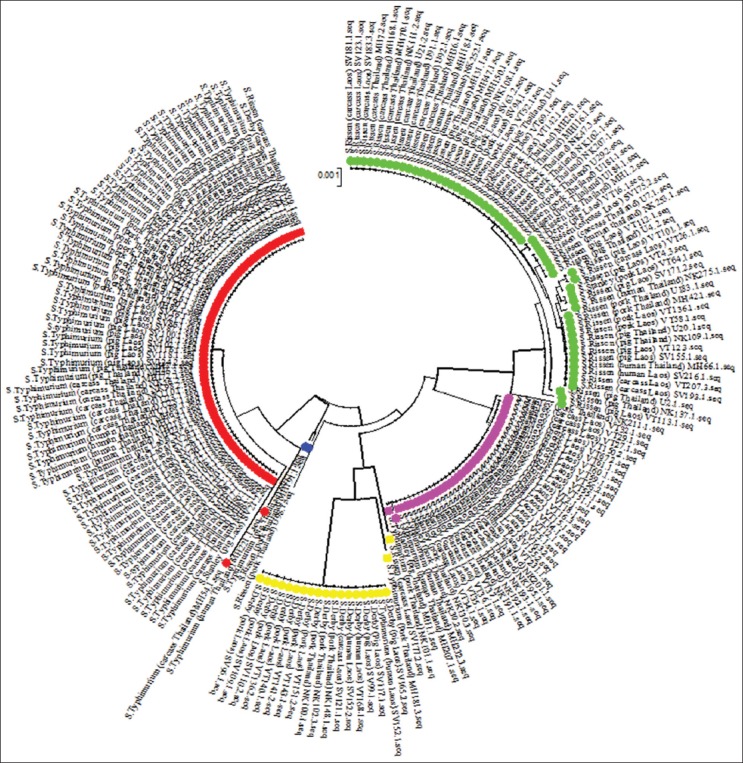
Phylogenetic relationships of 195 *Salmonella* isolates based on concatenated of the three MLST genes (*fimA*, *manB*, and *mdh*) sequenced were generated by MEGA6 using the neighbor-joining method. The tree is drawn to scale, with branch lengths in the same units as those of the evolutionary distances used to infer the phylogenetic tree. Each color represents different clusters (A - green, B - pink, C - yellow, D - blue, and E - red).

In this study, an NJ phylogenetic tree was constructed by concatenated *fimA, manB*, and *mdh* sequences (1989 bp). The tree showed that *Salmonella* isolates of four different serotype were separated into five clusters (Clusters A, B, C, D, and E) (Table-2). Members of the same serotypes clustered into distinct groups. The percentage of similarity within each cluster ranged from 99.6% to 100% and the average genetic distance of *Salmonella* isolates within each cluster ranged from 0.001 to 0.003 (Figure-1). This study examined *S. enterica* isolated from pig production and human to determine the genetic relatedness of four different serotypes from various hosts. 195 *Salmonella* isolates of four serovars (Typhimurium, Rissen, Derby, and Stanley) in Thailand–Laos border provinces were assessed using molecular typing based on the sequences of three highly conserved genes (*fimA, manB, and mdh*). The molecular typing schemes utilized the combination of housekeeping genes and virulence or flagellin genes and offered more discriminatory power in determining the evolutionary relationship of *S. enterica* serovars [18,19]. The four serotypes used in this study were commonly found in pig production and human in Thailand–Laos border provinces. They have been incriminated in several cases of salmonellosis in humans that occur due to the consumption of contaminated pork or ingestion of water contaminated by pig feces. Phylogenetic analysis revealed that *Salmonella* isolates were grouped into five different clusters and members of the same serotypes were found in the same cluster (Table-1 and Figure-1). This showed that Cluster A consists of 56 *S. enterica* serotype Rissen, including pig fecal sample (n=17), pig carcass (n=16), pork (n=17), and human isolates (n=6) from Thailand–Laos border provinces. Cluster B contained *S*. *enterica* ser. Stanley (n=34) and *S. enterica* ser. Typhimurium (n=3), Cluster C contained *S. enterica* ser. Derby (n=17), Cluster D contained one *S. enterica* ser. Rissen and one *S. enterica* ser. Typhimurium, and Cluster E contained *S*. ser. Typhimurium (n=73) (Table-2). Salmonella isolated from pig production and human in Thailand–Laos border provinces represents overlapping population. This is because consumption of contaminated pork and other pig product can increase the risk of salmonellosis in humans in addition to significant economic losses. Contamination of pork can occur during processing through improper handling of the intestinal content at the slaughterhouse [20]. This can, in turn, increase the chances of transmission of *Salmonella* serotypes from humans to animals and vice versa.

**Table-2:** Distribution of *Salmonella* serotype among pig, pig carcass, pork, and human isolates.

Cluster	*Salmonella* serovars	Sample type (Number of isolates)									

		Thailand					Laos				
	
		Pig	Pig carcass	Pork	Human	Total	Pig	Pig carcass	Pork	Human	Total
A	*S*. Rissen	10	9	10	5	34	7	7	7	1	22
	*S*. Typhimurium	1	0	0	0	1	0	0	0	0	0
	*S*. Derby	0	0	0	0	0	1	0	0	0	1
	*S*. Stanley	0	0	0	0	0	0	0	1	0	1
B	*S*. Stanley	1	5	3	0	9	9	7	8	1	25
	*S*. Typhimurium	0	0	1	2	3	0	0	0	0	0
C	*S*. Derby	0	0	3	0	3	3	1	8	2	14
	*S*. Typhimurium	0	0	1	0	1	0	0	0	1	1
	*S*. Rissen	0	0	0	0	0	0	1	0	0	1
D	*S*. Rissen	0	0	1	0	1	0	0	0	0	0
	*S*. Typhimurium	1	0	0	0	1	0	0	0	0	0
E	*S*. Typhimurium	15	13	10	5	43	11	13	6	0	30
	*S*. Derby	0	0	0	0	0	0	1	0	0	1
	*S*. Stanley	0	0	0	0	0	1	0	0	0	1
	*S*. Rissen	0	1	0	0	1	0	0	0	0	0

Most of pig production and human isolates in Thailand–Laos border provinces in each cluster showed a high degree of similarity, indicating close relationship among the isolates. Exceptions were *S. enterica* ser. Rissen (n=3), *S. enterica* ser. Typhimurium (n=6), *S. enterica* ser. Stanley (n=2), and *S. enterica* ser. Derby (n=2). Three *S. enterica* ser. Rissen were found in different branches and one *S. enterica* ser. Rissen was isolated from pig carcass in Laos clustered with *S. enterica* ser. Derby (Cluster C). In addition, one *S. enterica* ser. Rissen isolates in Thailand from pig carcass clustered with *S. enterica* ser. Typhimurium (Cluster E) and one *S. enterica* ser. Rissen isolates in Thailand from pork clustered with *S. enterica* ser. Typhimurium (Cluster E). Six *S. enterica* ser. Typhimurium were in different branches; one *S. enterica* ser. Typhimurium isolates in Thailand from pig clustered with *S. enterica* ser. Rissen (Cluster A). Three *S. enterica* ser. Typhimurium clustered with *S. enteric* a ser. Stanley (Cluster B), two of the isolates were from humans in Thailand, while the other is from pig carcass from Thailand. Two *S. enterica* ser. Typhimurium clustered with *S. enterica* ser. Derby (Cluster B) contained one isolate in Laos which was from human and one isolate in Thailand which was from pork, and one *S. enterica* ser. Typhimurium isolate in Thailand was from pig clustered with *S. enterica* ser. Rissen (Cluster D). In addition, two *S. enterica* ser. Stanley were found in different branches, one of these from pork isolated in Laos clustered with *S. enterica* ser. Rissen (Cluster A) and the other clustered with *S. enterica* ser. Typhimurium (Cluster E). Similarly, two other S. *enterica* ser. Derby were on different branches of the phylogenetic tree, one of these clustered with *S. enterica* ser. Rissen (Cluster A) a pig isolates from Laos and the other clustered with *S. enterica* ser. Typhimurium (Cluster E). The clustering of the isolates with similar genotypes from pig, pork, and human in both Thailand and Lao could be attributed to factors such as the environment, transportation, and cross-contamination during handling and processing of pork [21]. Altogether, the results also indicate that pig and pork may be a reservoir for the *Salmonella* serovars in humans. Similar studies among *Salmonella* isolates from pig, pork, and human have been reported in Germany, France, and Mexico [22-25]. The combination of molecular typing technique and epidemiological studies plays a pivotal role in authenticating the relationship between *Salmonella* strains isolated from different regions and sources. These approaches also helped in providing valuable information on various contamination routes and differentiate strains obtained from sporadic and outbreak cases [26].

## Conclusion

In this study, sequence of the three conserved housekeeping genes of *Salmonella* serotypes isolated from pigs, pork, and humans from Thailand and Laos border provinces showed a high degree of similarity except for few isolates that cluster on different branches of the phylogenetic tree. Hence, giving room for the likelihood of animal to human transmission, increased dissemination and maintenance of resistance determinants and the risk of human infection.

## Authors’ Contributions

RC conceived and designed the study. RP and SA collected the samples. RP and NS carried out the experiment. AAB and RP analyzed the data. RP and AAB wrote the first draft of the manuscript. RC revised the manuscript. All authors read and approved the final draft of the manuscript.
